# The Management of Labor in Cases of Minor Pelvic Deformity

**Published:** 1895-09

**Authors:** Joseph Eve Allen

**Affiliations:** Augusta, Ga., President of the Augusta Academy of Medicine; Professor of Obstetrics in the Medical College of Georgia


ATLANTA

### Medical and Surgical Journal.

VOL. XII. SEPTEMBER, 1895. No. 7.

LUTHER B. GRANDY, M.D., MANAGING EDITOR.

M. B. HUTCHINS, M.D., BUSINESS MANAGER.

COLLABORATORS:
H. V. M. MILLER, M.D., LL.D., VIRGIL O. HARDON, M.D., FLOYD W. McRAE, M.D.,
DUNBAR ROY, M.D., AND H. R. SLACK, M.D., PH.M.

### *ORIGINAL COMMUNICATIONS.*

### THE MANAGEMENT OF LABOR IN CASES OF MINOR PELVIC DEFORMITY.

BY JOSEPH EVE ALLEN, M.D., AUGUSTA, GA.,

President of the Augusta Academy of Medicine; Professor of Obstetrics in the Medical College of Georgia.

Great pelvic deformity is fortunately very rare among the women of the South. Diathetic diseases, such as rachitis, osteomalacia, etc., which seriously affect the nutrition and growth of the bones, do not often occur in this section, and here an obstetrician may practice a lifetime without encountering one of those terrible cases of dystocia due to this cause, which are so common in some of the countries of Europe.

Cases of minor pelvic contraction are, however, much more frequent than is generally supposed, and are yearly on the increase, owing to the influx of foreign immigration and the altered conditions of life with the negro since the war. In these cases the deformity not being sufficient to prevent the birth of a live child, but only enough to render the labor difficult, long, and dangerous, it is usually unrecognized, and the trouble is attributed to uterine inertia, rigidity, or some cause other than the right one.

The early diagnosis of the lesser forms of distortion is very important, for upon it directly depends the safety and well-being of both the mother and child, because by it is determined the line of treatment to be pursued, and often small differences in conformation make a great difference in the procedures necessary for delivery. The diagnosis is sometimes extremely difficult, for in these cases there is seldom anything about the physique of the woman to indicate her abnormal condition, and unless a most thorough examination is made it will altogether escape attention until irreparable damage has been done. Great malformation on the other hand at once attracts notice and plainly points out the method of treatment; hence, in such cases statistics show proportionately much better results.

Extended experience in hospital and private midwifery practice daily more firmly convince me of the great necessity for the careful examination by palpation and pelvimetry of every primiparous pregnant woman long before beginning of labor, in order to detect dangers and be ready to avert disasters. The object of this paper is to demonstrate the value of this routine practice by reference to the mechanism and management of labors made difficult by the slighter degrees of pelvic distortion, and to urge its adoption upon all physicians who are engaged in obstetric work.

No man can practice obstetrics scientifically who does not in each case under his care know, not only the form and capacity of the woman's pelvis, but also the presentation, position, and approximate size of the fetus that has to pass through it; for upon a correct estimate of these relations is founded all hope of success when assistance is required. It will be readily seen at what a great disadvantage the accoucheur is placed who, unskilled in modern methods of diagnosis, or who, failing to obtain the information thus afforded, is suddenly called upon to face some pressing emergency or formidable operation. Under such circumstances it is impossible for him to properly discharge the responsibility resting upon him, for he is forced to do not what is best, but what is most available at the time, and thus is jeopardized the welfare and the lives of the two beings who are entirely dependent upon his aid for safety and relief.

Pelvimetry enables us to choose at once the proper operation, when one is necessary, and the man is certainly deserving of the severest censure who, without resorting to it for guidance, would attempt to deliver with forceps in a case where this instrument was contraindicated, or who would delay the performance of a capital operation, when one was imperatively demanded, until maternal or fetal exhaustion made it either utterly hopeless, or robbed it of its best results. As Grandin says: "The keynote of scientific obstetrics to-day is election," and the selection of the time for and the methods of operating should be the result of deliberate judgment, based on precision in diagnosis. The same principles apply in obstetrics as in surgery, and the same brilliant results can always be attained if the operator in his work will be governed by the dictates of advanced science.

In the majority of cases a few measurements will furnish all the data needed, as it is only in very exceptional instances that the lengths of all the pelvic diameters are required. For taking these Collyer's pelvimeter is the best instrument, as it is light, accurate, and easy to handle.

The important external measurements are the intercristal, the interspinous, the external conjugate, and the transverse diameter of the inferior strait. These measurements can all be taken in a few moments without the slightest inconvenience to the woman, and the taking of them is a duty just as obligatory upon the obstetrician as urinalysis or any other duty that he is called upon to perform. The intercristal diameter is the distance between the iliac crests at the widest point, and is normally eleven and a quarter inches. The anterior interspinous diameter is the distance between the anterior superior spines of the ilia, and is generally between ten and ten and a half inches. The external conjugate or diameter of Bandelocque is measured from the depression below the spine of the last lumbar vertebra to the upper border of the symphysis pubis, and averages about seven and a half inches, though this varies with the adipose development of the subject. The posterior interspinous diameter is that between the posterior superior iliac spines, and is normally from three and a third to three and a half inches. The transverse diameter of the inferior strait is a line drawn from one ischial tuberosity to the other, and usually is about four and three-quarter inches. The diagonal conjugate is measured from the middle of the promontory of the sacrum to the lower edge of the symphysis pubis; it is about from four and three-quarters to five and a quarter inches. By deducting three and a quarter inches from the external and three-fifths of an inch from the diagonal conjugate, the length of the true conjugate diameter of the superior strait is arrived at. This is the most important diameter of all, for generally if the brim is large enough to admit the head, there is sufficient room for it to pass through the entire pelvis.

In the class of minor pelvic deformities are included all cases in which the conjugate diameter of the brim measures not less than three or over four inches. In such cases the birth of a living child of average size is possible, though the contraction so affects the course of labor as to require the intervention of art to accomplish the delivery. The lesser pelvic distortions are commonly of two kinds, viz.: Flat pelves and justo-minor pelves, which differ widely in etiology, in their effect upon the mechanism of labor, and in the methods of delivery required.

The flat pelvis is the most common, and is produced by the falling downward and forward of the sacrum, owing to relaxation of the iliac ligaments. This displacement only narrows the conjugate diameter of the brim without affecting any of the other pelvic measurements; therefore, in labor the difficulty is entirely with the engagement of the head, and after this has taken place the subsequent progress is comparatively easy. The diagnosis is made by finding that the external and diagonal conjugates are diminished, and that the posterior superior iliac spines are nearer together than normal and above the level of the sacral spines. The intercristal and anterior interspinous diameters are about the same length. The pelvis is symmetrical, and the vertebral column is not curved.

In flat pelves the fetal head always enters in the transverse diameter of the brim, because there is not space enough for it in either of the obliques. There is also usually a considerable amount of extension which brings the anterior fontanelle within easy reach of the examining finger, and which, if continued, ends in a brow or face presentation. The head always engages in a position of parietal obliquity, that is, with one parietal bone in advance of the other; this is known as Nægele's obliquity, because it was first pointed out by that distinguished obstetrician. The anterior lying parietal is always the lower, because the forward position of the sacral promontory prevents the descent of the posterior lying bone. The passage through the superior strait is effected by the anterior parietal becoming fixed against the pubis, and the posterior parietal rotating past the promontory often becoming grooved and dinted by it. Sometimes the posterior lying parietal bone is the lower; this form of obliquity is exceedingly unfavorable and is almost always followed by impaction if not promptly corrected. When the head does pass the brim in the position of posterior parietal obliquity, which can only happen with very small or premature children, it is either by the anterior parietal being driven down and the posterior moving up, or by the posterior parietal remaining stationary against the promontory and the anterior parietal turning round this point as a center of rotation. Litzmann has laid down a most excellent practical rule regarding labor in flat pelves which should ever be borne in mind in treating these cases, namely: that the distance between the sagittal suture and the sacral promontory is the true guide to the proportion between the size of the head and that of the pelvis, and that when this is not less than three-fourths of an inch delivery can usually be easily accomplished.

The justo-minor pelvis is one that is contracted in all of its diameters. There is, however, the same relation between the measurements as in the normal pelvis, thus the anterior interspinous varies from the intercristal diameter by about an inch. The obliques are the longest diameters of the brim and the antero-posterior is the longest of the outlet. The posterior iliac spines are on a level with the sacral spines and the ilio-pectineal line can be traced throughout its entire extent. The mechanism of labor in justo-minor pelves is the same as that in normal and very different from that in flat pelves. The difficulty is not only at the inlet but throughout the whole passage. The problem which confronts the practitioner is the same as that presented by labor in a well formed pelvis with an over-large head. The head always enters in one of the oblique diameters, and its flexion must be extreme in order to adapt the sub-occipito-frontal diameter to the contracted brim. Forward rotation of the occiput occurs early in justo-minor pelves, because the head meets with resistance from the bones as well as the soft parts, and hence the occiput has to turn well to the front so as to pass the outlet. Nægale's obliquity is never met with in this form of dystocia. In these cases posterior occipital positions of the vertex are especially bad.

All forms of pelvic distortion have clinically certain symptoms in common, which, when presented by a parturient woman, immediately suggest to the judicious accoucheur the fact of bony obstruction, and call for a rigid examination to determine its character and extent. In these cases during the first stage of labor there is greater mobility of the uterus and fetus than is natural, the contraction preventing that settling of the head into the brim which usually occurs during the last weeks of pregnancy. This increased mobility tends to produce lateral obliquity of the uterus, pendulous abdomen and malpresentations. The presentations also frequently change. The membranes generally rupture prematurely, because the head not coming down into the os, the mass of water is not dammed back, and hence the full intrauterine pressure is exerted against them. The amniotic sac bulges more than in ordinary labor, often protruding like a glove finger through the undilated os, and when the waters break prolapse of the cord is liable to happen. As the head does not fill up the lower uterine segment, after rupture of the membranes the liquor amnii all drains away and dry labor follows. During the second stage, when the head becomes engaged at the superior strait, the cervix is frequently caught between it and the pelvic bones and becomes enormously swollen and edematous, and the labor continuing, if assistance is not promptly afforded injurious pressure will be made upon the soft parts of the pelvis, or the violent ineffectual action of the uterus may result in rupture of that organ. An immense caput succedaneum forms upon the infant's head, and often a hematoma, due to capillary hemorrhage between the scalp and skull, occurs. The cranial bones greatly overlap one another at the sutures and are dinted and flattened by the pressure to which they are subjected. Violent, irregular, or tetanic contraction of the uterus is a positive indication of obstruction in the birth canal which demands immediate action on the part of the medical attendant.

Nothing in the whole range of his special practice devolves greater responsibility upon the obstetrician, calling for the fullest exercise of sound judgment, ripe experience, and superior operative skill, than the management of labor in cases of pelvic deformity. In such cases at full term, if the antero-posterior diameter of the superior strait is not less than three inches and the fetus of average size, with the vertex presenting, he has, between two operative procedures, to select that method of delivery which offers the least risk to mother and child. The choice between version and the forceps depends principally upon the degree of adaptability of the head to the brim. If in a flat pelvis the head can be pushed into the brim, or is so far engaged that the occipito-frontal diameter is just above it, the forceps should be applied and the attempt made to drag the head past the obstruction. If, however, the head does not easily engage, then podalic version had best be done as soon as the cervix is sufficiently dilated, for by turning, the head is brought through the pelvis base first, and thus the longest diameter to pass the superior strait is the bitemporal, which is three inches. The parietal bones being pressed together lengthens the vertical and shortens the transverse measurements of the head; whereas, with the vertex presenting, the resistance met tends to flatten the head, diminishing the vertical and increasing the biparietal measurement. Whenever in a flat pelvis the head presents with the occiput directed behind, or if there is much extension, or the face or brow presents, the child should be delivered by turning. If the vertex presents favorably, however, the advantage to the mother of turning over the forceps is very little and is counterbalanced by the danger to the child in extraction; consequently in such cases the forceps should be used.

In justo-minor pelves delivery by version should never be resorted to, as the infant will surely be sacrificed, for the entire pelvis is so contracted that fatal compression of the cord is inevitable.

In these cases, when the head does not come down into the brim, Taylor's forceps should be applied. This instrument has long and very narrow blades and can be used when the os is not much dilated. Its inventor did not intend that it should be used as a tractor, but simply as a means of holding the head flexed and in a favorable position against the cervix until full dilatation and engagement can be effected, after which this instrument is to be removed and a stronger one applied. In most cases, however, it will be found that Taylor's forceps are fully sufficient for all the purposes of extraction. When the head becomes impacted, forceps must, of course, be used at once. The evidences of impaction are unmistakable, but the obstetrician should not wait for their appearance before rendering instrumental assistance. If with strong pains the second stage of labor has lasted two hours, the forceps should be used, although edema of the vulva and vagina has not begun. When the head is high in the pelvis, traction should be made with the pains; when, however, it reaches the outlet, the pulling should be done between the pains.

The study of minor pelvic deformity in all of its relations proves that in flat pelves version is generally the best method of delivery, while in justo-minor pelves the forceps always subserves best the interests of both mother and child.

